# An individualized transcriptional signature to predict the epithelial-mesenchymal transition based on relative expression ordering

**DOI:** 10.18632/aging.103407

**Published:** 2020-07-08

**Authors:** Tingting Chen, Zhangxiang Zhao, Bo Chen, Yuquan Wang, Fan Yang, Chengyu Wang, Qi Dong, Yaoyao Liu, Haihai Liang, Wenyuan Zhao, Lishuang Qi, Yan Xu, Yunyan Gu

**Affiliations:** 1 College of Bioinformatics Science and Technology, Harbin Medical University, Harbin, China; 2 College of Pharmacy, Harbin Medical University, Harbin, China

**Keywords:** epithelial-mesenchymal transition, 16-gene pair signature, relative expression orderings, prognosis, immune checkpoint genes

## Abstract

The epithelial-mesenchymal transition (EMT) process is involved in cancer cell metastasis and immune system activation. Hence, identification of gene expression signatures capable of predicting the EMT status of cancer cells is essential for development of therapeutic strategies. However, quantitative identification of EMT markers is limited by batch effects, the platform used, or normalization methods. We hypothesized that a set of EMT-related relative expression orderings are highly stable in epithelial samples yet are reversed in mesenchymal samples. To test this hypothesis, we analyzed transcriptome data for ovarian cancer cohorts from publicly available databases, to develop a qualitative 16-gene pair signature (16-GPS) that effectively distinguishes the mesenchymal from epithelial phenotype. Our method was superior to previous quantitative methods in terms of classification accuracy and applicability to individualized patients without requiring data normalization. Patients with mesenchymal-like ovarian cancer showed poorer overall survival compared to patients with epithelial-like ovarian cancer. Additionally, EMT score was positively correlated with expression of immune checkpoint genes and metastasis. We, therefore, established a robust EMT 16-GPS that is independent of detection platform, batch effects and individual variations, and which represents a qualitative signature for investigating the EMT and providing insights into immunotherapy for ovarian cancer patients.

## INTRODUCTION

The epithelial-mesenchymal transition (EMT) is the transition of cells from an epithelial phenotype to mesenchymal [1]. Importantly, the EMT is associated with disease progression and poor prognosis in various cancers [2, 3], including ovarian cancer (OvCa). During the EMT process, epithelial cells lose their properties, including compact organization in colonies, and acquire a spindle-like morphology with enhanced migratory capacity. The hallmarks of the EMT include loss of the cell-cell adhesion protein E-cadherin (encoded by *CDH1*) and gain of the cytoskeletal protein vimentin (encoded by *VIM*) [4].

OvCa is a highly metastatic malignant gynecologic neoplasm. Most OvCa cases are diagnosed at advanced stages, resulting in an often poor prognosis [5]. As such, OvCa is the second leading cause of cancer-related death in women [6], largely resulting from the high metastatic potential of the cancer cells. Growing evidence suggests that metastasis of OvCa cells is accompanied by features of the EMT [1, 2]. Indeed, patients with OvCa that have cancer cells with mesenchymal status, have significantly worse outcomes [7, 8]. Additionally, the EMT status is positively correlated with expression of immune checkpoint genes in tumors [1], providing new insights into immunotherapy. Hence, it is necessary to identify robust EMT signatures in cancer patients to more accurately predict metastasis, immune responses, and prognosis.

Developing an EMT signature based on the expression values of EMT-related markers, such as cadherin 2 (*CDH2*), Forkhead box protein C2 (*FOXC2*), Snail family transcriptional repressor 1, fibronectin 1, and matrix metalloproteinase 2, has become a popular approach in current research [1]. Many additional EMT-inducing factors have also been implicated in prediction of EMT status, and represent potential prognostic signatures [9]. For instance, Gibbons et al. surveyed a 16-gene signature of canonical EMT markers in The Cancer Genome Atlas (TCGA) pancancer cohort, including *VIM*, *CDH2*, and *FOXC2* [8]. Moreover, Chae et al*.* conducted an integrated analysis of the immune landscape through EMT scores derived from the 16-gene signature [10]. Similarly, Mark et al*.* conducted an integrated, global analysis of genomic and proteomic profiles associated with the EMT across 1,934 tumors and developed a signature comprised of 77 unique EMT genes correlated with known core EMT markers across diverse tumor types [1]. Taube et al*.* defined a 249 gene expression signature derived from a meta-analysis of differential gene expression in breast cancer cell lines, which was found to be closely associated with the claudin-low and metaplastic breast cancer subtypes, and negatively correlated with the pathological complete response [11].

However, there are some limitations to the above quantitative expression-based algorithms when applied to independent cohorts as absolute gene expression values are susceptible to systematic biases related to batch effects, platform differences, and normalization methods. Currently, one of the most common solutions to this challenge is the normalization of data for adjusting the batch effect. However, this requires researchers to pre-collect sequencing information from a large number of patients, which is not feasible for individualized medicine. In addition, gene expression heterogeneity within individual samples may increase the noise associated with the data, thus hindering the application of the EMT signature. Therefore, a robust signature to efficiently distinguish the EMT status in individual patients is urgently needed.

Within-sample relative expression orderings (REOs) of genes are stable in some cancer types, such as in primary cancer tissues, however, are typically reversed in the corresponding metastatic cancer tissues [12]. This biological phenomenon establishes a basis for analyses based on REOs of gene pairs to characterize cancer subtypes [13, 14]. Further, the within-sample REOs between genes have been shown to be robust against interindividual biological variations and batch effects, RNA degradation, specimen preparation, and data normalization [15]. Hence, specific methods have been successfully developed to identify prognostic, drug response, and subtype specific signatures in various cancers based on within-sample REOs [12, 16, 17].

In this study, we hypothesized that a set of REOs between genes exists and is highly stable in patients with epithelial-type cancer, while showing the opposite effect in patients with mesenchymal-type cancer, enabling use of this gene set as a predictive signature for EMT status.

## RESULTS

### Identification of EMT-associated differentially expressed genes (DEGs)

We collected data from three cohorts with EMT labels from TCGA (n = 459) to identify EMT-associated genes, as shown in Table 1. Using TCGA data, we identified 5695 DEGs between 287 epithelial OvCa samples and 172 mesenchymal OvCa samples with an FDR of less than 0.05. Among the DEG lists, 1966 DEGs were upregulated and 3729 DEGs were downregulated in mesenchymal OvCa samples compared with epithelial OvCa samples.

**Table 1:** Gene expression profiles of OvCa used in our study.

**Accession**	**EMT status**		**All samples**	**Platform**
	**Epithelial**	**Mesenchymal**		
**Datasets for developing the REO-based 16-GPS (training dataset)**				
TCGA	287	172	459	Affymetrix U133A
**Datasets for validating the performance of the signature (validation dataset)**				
GSE9891	140	102	242	Affymetrix U133APlus2
GSE26712	102	83	185	Affymetrix U133A
GSE49997	-	-	194	ABI HGSM Version 2
ICGC	-	-	93	Illumina HumanOmni25-8
GSE52999	-	-	6	Illumina HumanHT-12 V30
GSE73168	-	-	16	Affymetrix U133APlus2
GSE18549	-	-	14	Affymetrix U133APlus2
GSE30587	-	-	18	Affymetrix Human Gene 10
GSE63885	-	-	101	Affymetrix U133APlus2

### REO-based identification of the EMT signature

**Figure 1:**
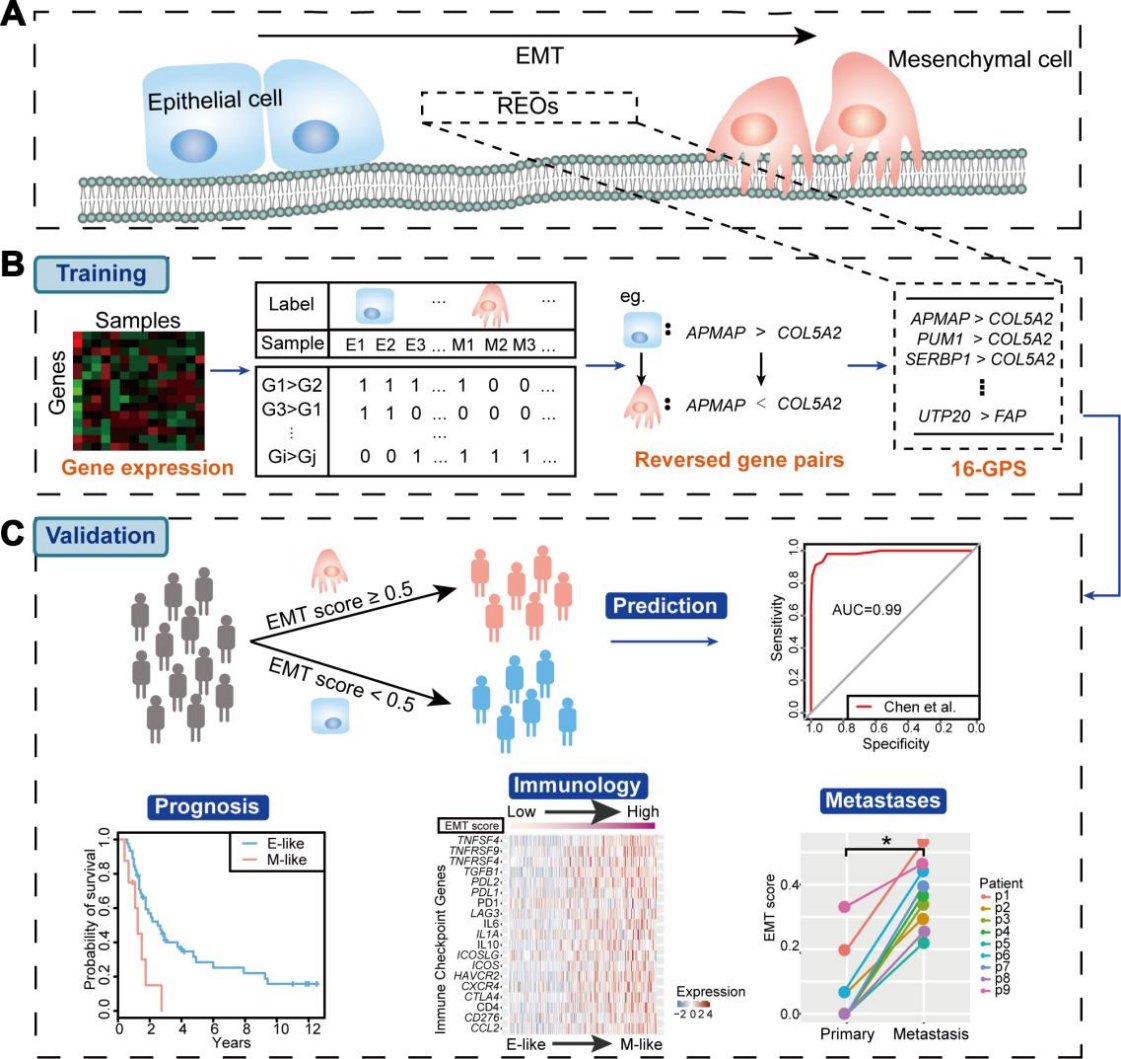
**Workflow of our study.** (**A**) Schematic diagram of the EMT. (**B**) Identification of the EMT signature (16-GPS). (**C**) Validation of the 16-GPS. In the independent validation cohorts, patient samples were classified as epithelial-like (E-like) or mesenchymal-like (M-like). The utility of 16-GPS was then validated by evaluating classification accuracy with ROC curves and associations with prognosis, immunology, and metastases.

**Figure 2:**
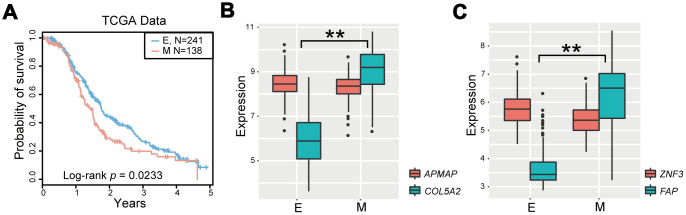
**Identification of the EMT signature for OvCa.** (**A**) Kaplan-Meier survival curves according to OvCa epithelial status and mesenchymal status in TCGA cases treated with platinum-based therapies. (**B**, **C**) Boxplot of expression levels of two gene pairs (APMAP and COL5A2, and ZNF3 and FAP). *P < 0.05, **P < 0.01 in Wilcoxon rank-sum test. E, epithelial status; M, mesenchymal status.

To identify qualitative EMT signatures, we performed gene expression analysis based on the REO strategy. The discovery workflow is briefly described in Figure 1. First, we utilized the TCGA OvCa cohort as the training dataset, which included expression levels of 11621 mRNAs in 459 samples, including 287 epithelial OvCa samples and 172 mesenchymal OvCa samples, of which 98% (169 of 172) of patients with mesenchymal-type OvCa had advanced-stage disease (stages III and IV). Moreover, patients with mesenchymal-type OvCa had significantly poorer overall survival than patients with epithelial-type OvCa in TCGA cohorts (*P* = 0.0233, log-rank test, Figure 2A). Our findings were consistent with previous studies [18], suggesting that patients with mesenchymal status tend to have poorer overall survival.

Next, in the TCGA dataset, we identified 31,278,124 gene pairs with stable REOs in more than 99% of epithelial OvCa samples. Among the stable gene pairs, 375,819 had significantly reversed REOs in mesenchymal samples (*P* < 0.05, Fisher’s exact test). After optimization of data with expression reversal ratios of greater than 75% and gene pairs including at least one EMT-associated DEG, we obtained a list of 16 gene pairs (16-GPS; Table 2) containing 18 genes, which we defined as an EMT-associated signature.

The gene pair with EMT-associated DEGs (collagen type V α 2 chain [*COL5A2*], fibroblast activation protein α [*FAP*]) is shown in Figure 2B and 2C. *COL5A2* and *FAP* were significantly upregulated in mesenchymal OvCa samples compared with epithelial OvCa samples (*P* = 8.43E-63 for *COL5A2* and *P* = 3.05E-61 for *FAP*, Wilcoxon rank-sum test).

**Table 2:** EMT-related 16-GPS.

**16-GPS^a^**	**REOs (G_*i*_ < G_*j*_)^b^**	***P* value^c^**	**Reversal ratio^d^**
Gene pair 1	*APMAP < COL5A2*	4.22E-83	80.23%
Gene pair 2	*PUM1 < COL5A2*	1.23E-76	77.33%
Gene pair 3	*SERBP1 < COL5A2*	9.85E-76	76.74%
Gene pair 4	*HNRNPR < COL5A2*	7.80E-75	75.00%
Gene pair 5	*TRIP12 < COL5A2*	5.92E-74	75.58%
Gene pair 6	*SHFM1 < COL5A2*	4.44E-73	75.00%
Gene pair 7	*SULT1A2 < FAP*	2.09E-79	79.07%
Gene pair 8	*EXOSC2 < FAP*	2.50E-79	77.91%
Gene pair 9	*ZNF20 < FAP*	1.79E-78	78.49%
Gene pair 10	*EWSR1 < FAP*	1.50E-77	77.91%
Gene pair 11	*SMCHD1 < FAP*	1.23E-76	77.33%
Gene pair 12	*DNAJC8 < FAP*	1.03E-75	75.58%
Gene pair 13	*TAF12 < FAP*	7.71E-75	76.16%
Gene pair 14	*ZNF3 < FAP*	7.71E-75	76.16%
Gene pair 15	*BCL7B < FAP*	7.71E-75	76.16%
Gene pair 16	*UTP20 < FAP*	5.92E-74	75.58%

^a^ Determination rule: a sample was determined to be epithelial-like if more than half of the gene pairs were consistent with the REOs (G_*i*_ < G_*j*_); otherwise, the sample was considered mesenchymal-like.

^b^ Represents the stable REO pattern (G_*i*_ > G_*j*_) in epithelial samples, but with a reversed REO pattern (G_*i*_ < G_*j*_) in mesenchymal samples.

^c^ *P* values were calculated by Fisher’s exact test. Gene pairs with *P* values of less than 0.05 were defined as significantly reversed gene pairs.

^d^ Reversal ratio denotes the ratio of the gene pair reversed in mesenchymal samples.

### Comparison of the 16-GPS with other EMT signatures

**Figure 3:**
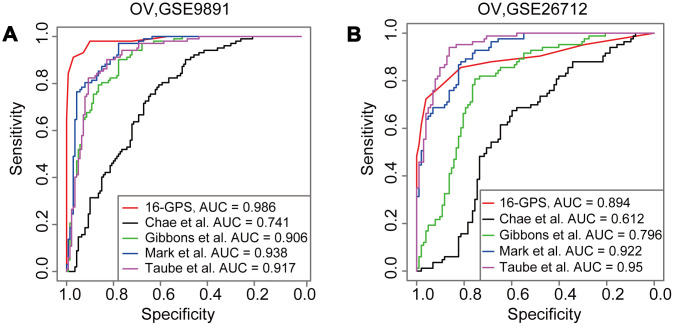
**Comparison of AUCs derived from the 16-GPS and other EMT signatures.** (**A**, **B**) ROCs derived from the signatures reported by Chae et al*.* (black line), Gibbons et al*.* (green line), Mark et al*.* (blue line), Taube et al*.* (purple line), and the 16-GPS (red line) in the GSE9891 (**A**) and GSE26712 datasets (**B**).

Several published EMT signatures based on the quantitative expression levels and kinetics of epithelial and mesenchymal cells have been reported (Supplementary Table 1) [1, 8, 10, 19]. The highest classification accuracy was achieved by the 16-GPS (AUC = 0.986) in the GSE9891 dataset (Figure 3A). In addition, the third highest classification accuracy was achieved by the 16-GPS (AUC = 0.894) in the GSE26712 dataset (Figure 3B). Overall, our method was superior to other methods in terms of classification accuracy and applicability in individualized diagnosis without data normalization.

### Prognostic performance using the OvCa validation cohorts

**Figure 4:**
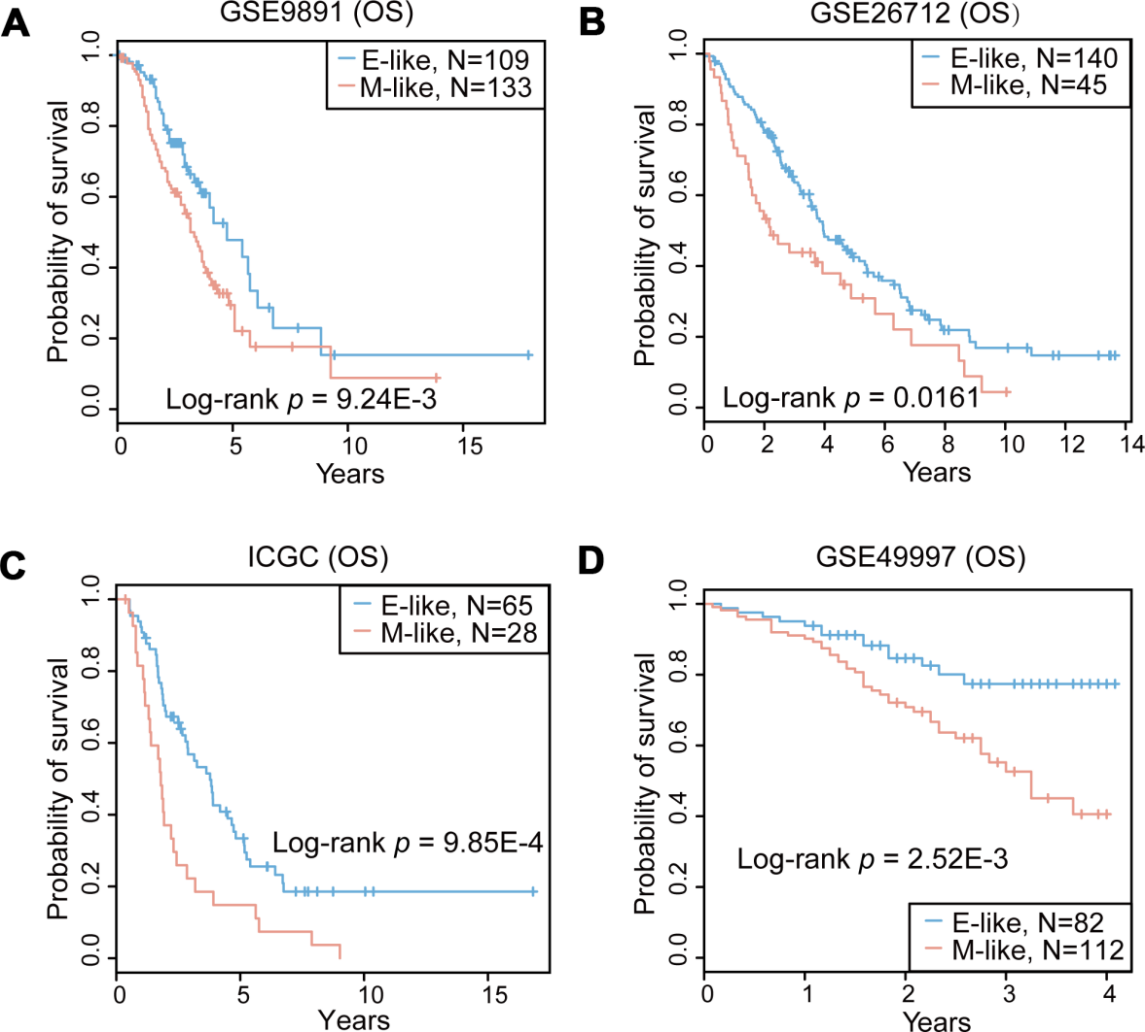
**Prognostic performance of the 16-GPS in the OvCa cohorts.** (**A**–**D**) Kaplan-Meier overall survival (OS) curves for the OvCa epithelial-like (E-like, blue) and mesenchymal-like (M-like, orange) phenotypes in GSE9891 (**A**), GSE26712 (**B**), ICGC (**C**), and GSE49997 (**D**) datasets.

The prediction efficiency of 16-GPS was tested in four independent OvCa datasets detected by various microarray platforms from different laboratories. According to the classification guidelines described above, patients with mesenchymal-like OvCa, as classified by 16-GPS, had significantly poorer overall survival than patients with epithelial-like OvCa (*P* = 9.24E-3 for GSE9891 and *P* = 0.0161 for GSE26712, log-rank test; Figure 4A and 4B), indicating that the 16-GPS could distinguish patients who may benefit most from clinical treatment. Moreover, ROC curves suggest that the classification accuracies in the two cohorts reached 0.99 for GSE9891, and 0.89 for GSE26712 (Figure 3A and 3B). We then used the 16-GPS to stratify patients with OvCa from two cohorts without EMT labels (ICGC and GSE49997) and observed that overall survival was significantly worse in patients with mesenchymal-like OvCa than in those with epithelial-like OvCa (*P* = 9.85E-4 for ICGC and *P* = 2.52E-3 for GSE49997, log-rank test; Figure 4C and 4D).

### Correlation between immune checkpoint expression and mesenchymal-like samples

**Figure 5:**
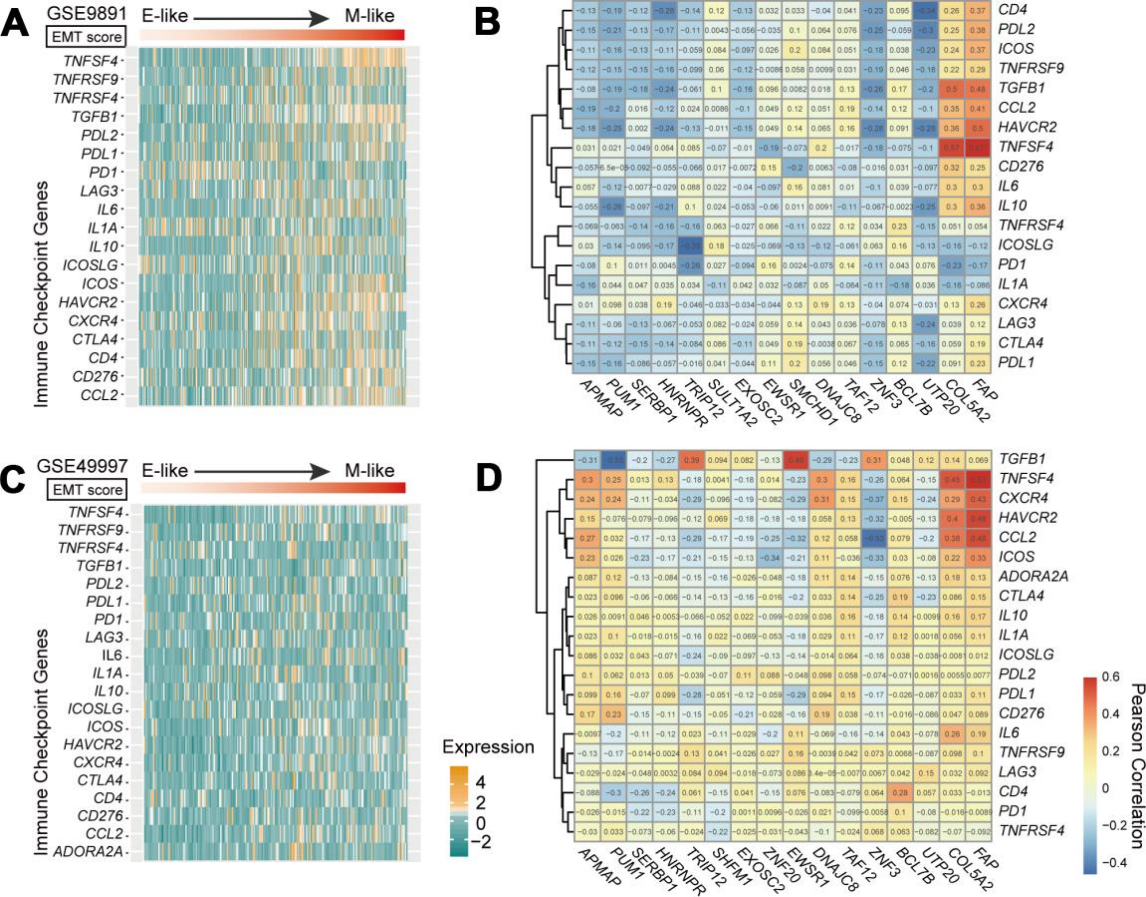
**Immune checkpoint genes in the EMT.** (**A**, **C**) Heatmap of mRNA expression levels of 20 immune checkpoint genes in GSE9891 (**A**) and GSE49997 (**C**) OvCa datasets. Tumor samples within each dataset were ordered according to EMT score (upper panels). (**B**, **D**) Correlations of expression levels between 18 genes in the 16-GPS and 20 immune checkpoint-related genes in the GSE9891 (**B**) and GSE49997 (**D**) OvCa datasets, tested by Pearson correlation analyses. Rows represent immune checkpoint genes, and columns represent genes in the 16-GPS.

Previous studies have reported that EMT is associated with suppression of antitumor immunity and is involved in immunotherapeutic mechanisms [21]. Therefore, we performed an expression analysis to investigate the relationship between the EMT and immune-related genes. We selected 20 potentially targetable immune checkpoint genes based on clinical trials or current drug inhibitors approved by the United Stated Food and Drug Administration [1] (Supplementary Table 2). We observed a significant positive correlation between EMT scores and the expression of immune checkpoint genes in GSE9891 dataset. The expression of immune targets was enriched in mesenchymal-like samples (Figure 5A). Moreover, we also assessed correlations in expression level between genes in the 16-GPS and immune-related genes. *COL5A2* and *FAP* were positively co-expressed with immune-related genes, including tumor necrosis factor superfamily member 4, *TGFB1*, C-C motif chemokine ligand 2, and hepatitis A virus cellular receptor 2 (median r = 0.43, *P* < 1.00E-9 for *COL5A2*; median r = 0.49, *P* < 1.00E-9 for *FAP*; Pearson correlation test; Figure 5B). In addition, programmed death ligand 1 (*PDL1*), *PDL2*, and cytotoxic T lymphocyte-associated protein 4 (*CTLA4*) showed higher expression levels in mesenchymal-like samples than in epithelial-like samples in the GSE9891 dataset (*P* < 0.05, Wilcoxon rank-sum test; Supplementary Figure 1A). Similar results were observed in the GSE26712 dataset (higher *PDL1* and *CTLA4* expression levels in mesenchymal-like samples, *P* < 0.05, Wilcoxon rank-sum test; Supplementary Figure 1B). In general, higher expression levels of immune-related genes were observed in mesenchymal-like samples than in epithelial-like samples. Moreover, we observed similar results in the OvCa cohort of the GSE49997 dataset (Figure 5C and 5D).

### Metastatic OvCa samples had high EMT scores

**Figure 6:**
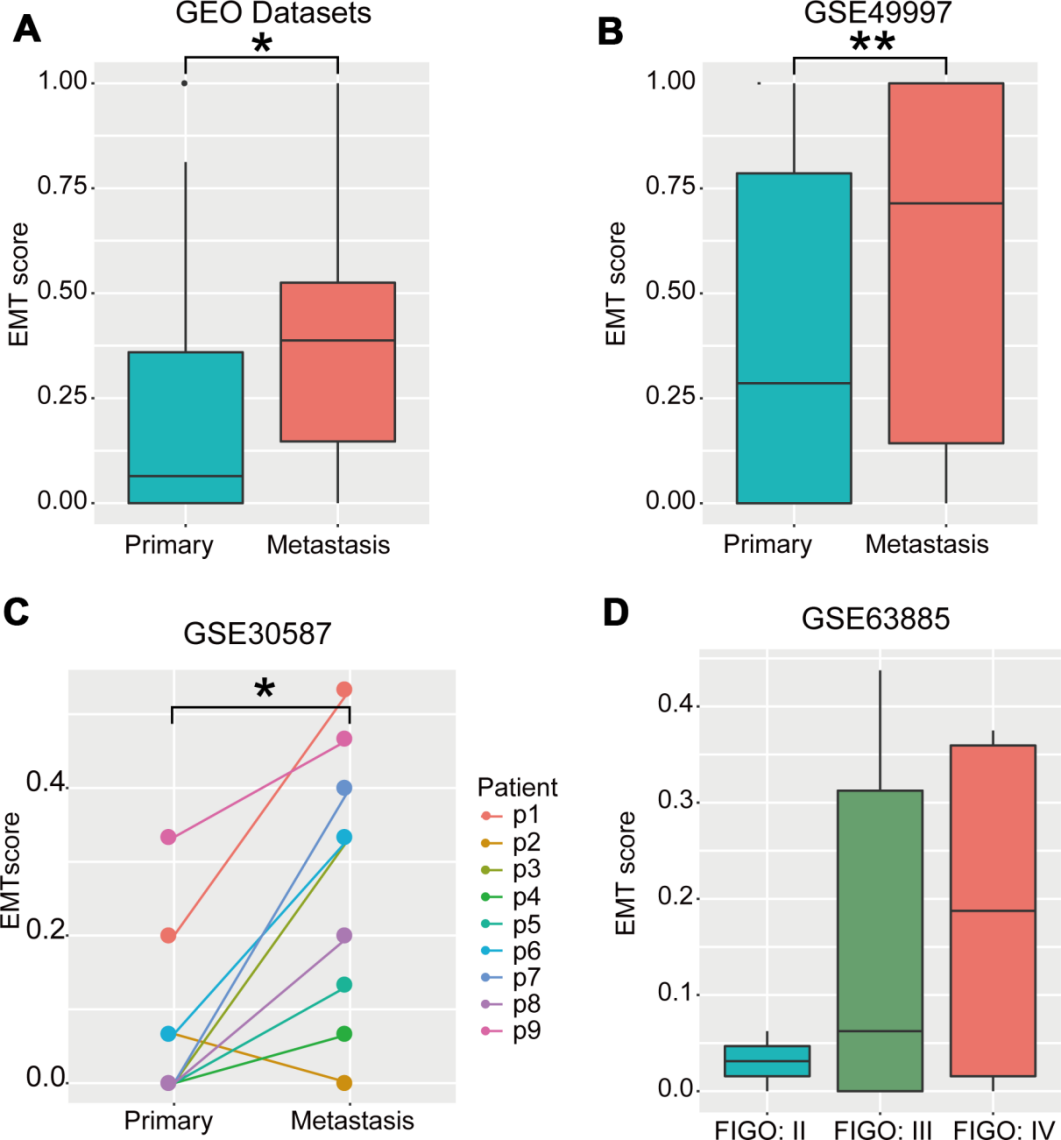
**EMT scores were negatively correlated with metastasis.** (**A**) Boxplot of EMT scores of primary and metastatic OvCa samples integrating the GSE52999, GSE73168, GSE18549, and GSE30587 datasets. (**B**) Boxplot of EMT scores in primary and metastatic OvCa samples (GSE49997). (**C**) Line chart of EMT scores in pair-wise primary and metastatic OvCa samples (GSE30587). (**D**) Boxplot of EMT scores in samples with different FIGO stages in OvCa (GSE63885). * *P* < 0.05, **P < 0.01 tested by Wilcoxon rank-sum test.

The EMT is important in promoting invasion and metastasis of cancer cells [1, 2]. A comparison of EMT scores between primary and metastatic tumors revealed that EMT scores of metastatic OvCa samples were significantly higher than those of primary OvCa samples (*P* < 0.05, Wilcoxon rank-sum test; Figure 6A and 6B). We further corroborated our results in a set of pair-wise samples, among which eight of nine (89%) cases exhibited significantly increased EMT scores in metastatic samples compared with those in primary samples for the same OvCa patients (*P* < 0.05, Wilcoxon rank-sum test; Figure 6C). Lastly, EMT scores were positively correlated with FIGO stage (Figure 6D), which is a system for histopathological diagnosis of tumor distribution, invasion, and metastasis representing the degree of cancer cell metastasis.

## DISCUSSION

In this study, we developed a qualitative transcriptomic signature (16-GPS) to predict EMT status in patients with OvCa based on within-sample REOs. The prediction efficiency of the 16-GPS outperformed other signatures. Notably, within independent OvCa cohorts, patients with mesenchymal-like OvCa, predicted by 16-GPS, showed poorer prognosis than those with epithelial-like OvCa following administration of platinum-based adjuvant chemotherapy. Additionally, patients with metastatic OvCa had higher EMT scores, confirming the reliability of the 16-GPS. Finally, our results showed that immune checkpoint-related genes tend to be upregulated in mesenchymal-like OvCa.

The 16-GPS contains two EMT-associated DEGs (*COL5A2*, *FAP*), which were among the top 10 genes overexpressed in the fibroblastic signature [21, 22]. Moreover, *COL5A2* and *FAP* are known EMT-associated genes that are included in the upregulated EMT core signature. Notably, *COL5A2* regulates the assembly of heterotypic fibers by encoding an α chain for a low abundance fibrillar collagen molecule. As a mesenchymal marker, *COL5A2* is upregulated in mesenchymal-like tumors [21]. A previous study showed that inhibition of FAP expression reduces cell adhesion, migration, invasion, and metastatic capacity, while inducing EMT in oral squamous cell carcinoma [22]. However, we were unable to determine whether *COL5A2* and *FAP* were highly expressed in individual patients. Nevertheless, based on REOs, we were able to identify the reference genes paired with *COL5A2* or *FAP*, which could be applied to individual patients as the EMT signature.

Importantly, the qualitative characteristics of the within-sample REOs increased the robustness of the approach against experimental batch effects from different laboratories and platforms, variations in the purity of tumor cells in tumor specimens, and RNA degradation during specimen preparation and sequencing. In addition, the REO method avoids the influence of bias in PCR micro-amplification, and can be applied in the clinical setting without the requirement of pre-collecting sample sets and normalization.

In this study, compared with other quantitative signatures [1, 8, 10, 19], although the 16-GPS did not achieve the most accurate classification in all validation datasets (Figure 3A and 3B), it was applicable to individual cancer patients to determine EMT status and predict prognoses. The other four signatures, which required pre-collection of sample sets for normalization and elimination of batch effects, could not be applied to individual samples. For instance, the method described by Da Yang et al*.* is dependent on a large number of cases and, therefore, cannot be applied to individual samples [23]. Most importantly, 16-GPS can predict the EMT status of individual cancer samples by detecting the expression levels of an 18-gene panel. Hence, this approach warrants more in-depth evaluation in further studies.

Correlation analysis between the expression levels of immune checkpoint-related genes and EMT scores showed that certain immune checkpoint genes were significantly overexpressed in mesenchymal-like OvCa samples. This finding is consistent with those of immunotherapy trials, which demonstrated higher sensitivity of immune checkpoint inhibitors in mesenchymal cancer cells than in epithelial cancer cells [1]. Save for *PD1* in GSE9891, and *PDL2* in GSE49997, the immune-related genes *PD1*, *PDL1*, *PDL2*, and *CTLA4* tended to be upregulated in mesenchymal OvCa samples compared with epithelial OvCa samples based on the rank of gene expression within each sample. Hence, specific factors other than those associated with EMT, such as sodium in the microenvironment or sex hormones, may also affect the immune response [24, 25].

Nevertheless, there were some limitations to the REO-based approach. The stable gene pairs were defined according to a predetermined percentage (e.g., 99%) in our study, and this threshold is flexible. Notably, strict control of the percentage could be beneficial for identifying more reliable gene pairs. During the two-step optimization, the reversal ratio was required to be greater than 75% in mesenchymal samples; this threshold was selected to expand the coverage of mesenchymal samples by reversed gene pairs. In addition, in our analysis of the correlation between metastasis and EMT score, one of the samples scored abnormally, which may have been caused by incomplete detection of genes in 16-GPS by the sequencing platform. Although Da Yang et al*.* classified TCGA OvCa samples as mesenchymal or epithelial labels based on an integrated genomic analyses of miRNA-regulatory network [23], some cancer patients have an epithelial-mesenchymal mixed status, which is defined as an intermediate status [8]. In our future studies, we will, therefore, aim to include patients with this intermediate status in our prediction using a continuous EMT scoring approach rather than binary categories. EMT is also involved in cancer progression and metastasis of other epithelial cancer types, such as lung cancer, breast cancer, liver cancer and so on [1, 26, 27]. We have tried to test the predictive performance of 16-GPS in lung cancer. Comparison of EMT scores between mesenchymal and epithelial lung cancer cell lines confirmed that EMT scores in mesenchymal cells were significantly higher than in epithelial cells (*P* = 0.0013 for GSE66616 and *P* = 0.00043 for GSE28709, Wilcoxon rank-sum test). We had predicted the EMT status of TCGA lung adenocarcinoma patients with administration of platinum-based adjuvant chemotherapy. However, overall survival between mesenchymal-like patients and epithelial-like patients did not show significant difference (*P* = 0.529, Wilcoxon rank-sum test). In our following study, we will explore the prediction and prognostic efficacy of 16-GPS in other cancers, such as breast cancer and liver cancer.

In summary, we developed an REO-based signature consisting of 16 gene pairs to predict the EMT status for OvCa patients. This approach was highly robust against experimental batch effects and is applicable to individual patients. The 18-gene panel may, therefore, be effective in predicting patients with OvCa who may benefit from platinum-based treatment. In the future, it may be possible to design a reverse transcription PCR kit to compare the expression levels of each gene pair in individual patients; however, this approach must be further validated in clinical trials. Moreover, we plan to apply the 16-GPS to other cancer types, including breast and lung cancer.

## MATERIALS AND METHODS

### Data and preprocessing

Gene expression data from patients with OvCa were collected from TCGA data portal, the Broad Institute’s GDAC Firehose (http://gdac.broadinstitute.org/), the Gene Expression Omnibus (GEO, http://www.ncbi.nlm.nih.gov/geo/) and Australia project in International Cancer Genome Consortium (ICGC, https://icgc.org/). The OvCa cohort (n = 459), downloaded from TCGA data portal, had sufficient clinical information and was evaluated using the Affymetrix U133A platform for training the EMT signature. For 80 samples in which records of platinum chemotherapy were omitted, samples were excluded from survival analysis. The EMT status of TCGA OvCa, GSE9891 and GSE26712 datasets was defined by Da Yang et al*.* [23]. Independent cohorts for validation were collected from GEO and ICGC (Table 1).

All probe-level mRNA profiles were annotated to Entrez gene IDs using R software. If a gene was mapped to multiple probe sets, the expression value for the gene was generated by calculating the average values. If a probe was mapped to multiple genes, the probe was excluded from subsequent analysis. Level 3 Affymetrix gene expression data were obtained from GDAC (http://gdac.broadinstitute.org/runs/stddata__2016_01_28/data/OV/20160128/).

### Identification of EMT-related differentially expressed genes (DEGs)

DEGs between epithelial and mesenchymal OvCa samples were determined by Wilcoxon rank-sum test based on the rank of gene expression within each sample. The *P* values were adjusted by the Benjamini-Hochberg (BH) procedure for multiple testing to control the false discovery rate (FDR). Genes with FDRs less than 5% were defined as DEGs. Subsequently, DEGs (epithelial versus mesenchymal) were identified from TCGA based on the rank of expression. The expression of these genes was postulated to be significantly associated with EMT status.

### Definition of stable and reverse gene pairs

Within-sample REO was defined as a binary relationship between gene *i* and gene *j*, where G_*i*_ > G_*j*_ or G_*i*_ < G_*j*_; G_*i*_ and G_*j*_ denote the expression values of gene *i* and gene *j*, respectively. The EMT status (epithelial type or mesenchymal type) was also a binary value. For a gene pair (G_*i*_ and G_*j*_), we defined the REO pattern of G_*i*_ > G_*j*_ as a stable REO if the number of samples with G_*i*_ > G_*j*_ accounted for more than 99% of the total number of epithelial samples.

In our study, we hypothesized that there was a set of EMT-related REOs that were highly stable in epithelial samples but reversed in mesenchymal samples (Figure 1A). In TCGA discovery dataset, we defined the reversed gene pair with a stable REO pattern (G_*i*_ > G_*j*_) in more than 99% of epithelial samples, however, with a significantly reversed REO pattern (G_*i*_ < G_*j*_) in mesenchymal samples tested by Fisher’s test with *P* values of less than 0.05. Finally, these reversed gene pairs were chosen as candidates for subsequent screen of an EMT signature (Figure 1B).

### Development of EMT signatures

EMT signatures were generated from two-step optimization of the candidate reversed gene pairs. First, reversed gene pairs consisting of at least one EMT-associated DEG were extracted. Next, the reversal ratio of gene pairs in mesenchymal samples was required to be more than 75%. The EMT score was calculated for each OvCa sample using the following formulas:

$$Rank\;Difference=\left[\begin{array}{c} G_{i1}\\ G_{i2}\\ \cdots \\ G_{in} \end{array}\right]-\left[\begin{array}{c} G_{j1}\\ G_{j2}\\ \cdots \\ G_{jn} \end{array}\right]$$ (1)

$$EMT\;Score=\frac{NUM(values\;<\;0),\;in\;Rank\;Difference}{NUM(gene\;pairs),\;in\;EMT\;signature},\;ranges\;from\;0\;to\;1.$$ (2)

$$Determination=\left(\begin{array}{c} EMT\;Score\geq 0.5,\;mesenchymal-like\;group\\ EMT\;Score<0.5,\;epithelial-like\;group \end{array}\right)$$ (3)

where rank difference denotes the difference between the ranks of two genes within each pair (1). For one OvCa sample, EMT score was calculated as the proportion of gene pairs (G_*i*_ < G_*j*_) with values of rank differences less than 0 among the total gene pairs of the EMT signature. The EMT signature identified in our study included 16 gene pairs (n = 16) (2). According to (3), samples were divided into the mesenchymal-like group (EMT score ≥ 0.5) or epithelial-like group (EMT score < 0.5).

### Survival analysis

Kaplan-Meier survival curves for survival analysis were calculated by log-rank test. Results with log-rank *P* values of less than 0.05 were considered significant. Patients who died during follow-up were censored.

### Statistical analysis

The area under the curve (AUC) of the receiver operating characteristic (ROC) curve was used to assess binary classification performance of the signature. Statistical analyses were performed with the Wilcoxon rank-sum test for 2-group comparisons. A value *P* < 0.05 was considered statistically significant. All statistical computations in this study were carried out using R software version 3.5.0 (http://www.r-project.org/).

## Supplementary Material

Supplementary Figure 1

Supplementary Table 1

Supplementary Table 2
