# Supplementary material for: An individualized transcriptional signature to predict the epithelial-mesenchymal transition based on relative expression ordering
Source: Aging (Albany NY). 2020 Jul 8;12(13):13172–86. doi: 10.18632/aging.103407 (PMC7377874; doi:10.18632/aging.103407)
Supplement: Supplementary Figure 1 [file aging-12-103407-s003..pdf]

SUPPLEMENTARY FIGURE

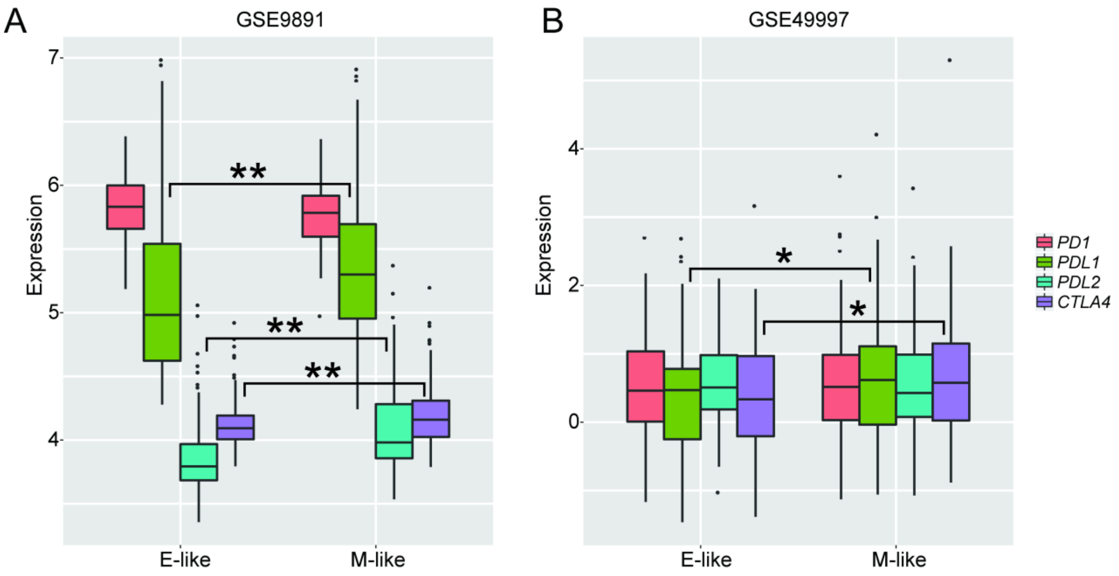

Supplementary Figure 1. Expression of four immune targets. (**A**, **B**) Comparison of expressions of four immune targets (PD1, PDL1, PDL2, CTLA4) between epithelial-like (E-like) and mesenchymal-like (M-like) in GSE9891 (**A**) and GSE49997 (**B**) OvCa datasets. Statistical significance at *P<0.05,**P<0.01 tested by Wilcoxon rank-sum test.
