# Supplementary material for: An individualized transcriptional signature to predict the epithelial-mesenchymal transition based on relative expression ordering
Source: Aging (Albany NY). 2020 Jul 8;12(13):13172–86. doi: 10.18632/aging.103407 (PMC7377874; doi:10.18632/aging.103407)
Supplement: Supplementary Table 1 [file aging-12-103407-s001..docx]

### Supplementary Table 1. Four published EMT-signature.

| **Chae2018** | | | **Gibbons2017** | | | **Mark2016** | | | **Taube2015** | | |
| --- | --- | --- | --- | --- | --- | --- | --- | --- | --- | --- | --- |
| Id | Symbol | Marker | Id | Symbol | Marker | Id | Symbol | Marker | Id | Symbol | Marker |
| 7431 | VIM | M | 7431 | VIM | M | 8038 | ADAM12 | M | 55857 | C20ORF19 | E |
| 1000 | CDH2 | M | 1000 | CDH2 | M | 81792 | ADAMTS12 | M | 29887 | SNX10 | E |
| 2303 | FOXC2 | M | 2303 | FOXC2 | M | 9509 | ADAMTS2 | M | 8626 | TP73L | E |
| 6615 | SNAI1 | M | 6615 | SNAI1 | M | 165 | AEBP1 | M | 3775 | KCNK1 | E |
| 6591 | SNAI2 | M | 6591 | SNAI2 | M | 23452 | ANGPTL2 | M | 624 | BDKRB2 | E |
| 7291 | TWIST1 | M | 7291 | TWIST1 | M | 84168 | ANTXR1 | M | 653145 | ANXA8 | E |
| 2335 | FN1 | M | 2335 | FN1 | M | 164 | AP1G1 | E | 728113 | ANXA8L1 | E |
| 3694 | ITGB6 | M | 3694 | ITGB6 | M | 5205 | ATP8B1 | E | 79651 | RHBDF2 | E |
| 4313 | MMP2 | M | 4313 | MMP2 | M | 558 | AXL | M | 653562 | SLC6A10PB | E |
| 4314 | MMP3 | M | 4314 | MMP3 | M | 54796 | BNC2 | M | 386757 | SLC6A10P | E |
| 4318 | MMP9 | M | 4318 | MMP9 | M | 800 | CALD1 | M | 3875 | KRT18 | E |
| 6663 | SOX10 | M | 6663 | SOX10 | M | 999 | CDH1 | E | 1040 | CDS1 | E |
| 145258 | GSC | M | 2729;7357 | GCS | M | 1000 | CDH2 | M | 7056 | THBD | E |
| 999 | CDH1 | E | 999 | CDH1 | E | 1040 | CDS1 | E | 4741 | NEFM | E |
| 1832 | DSP | E | 1832 | DSP | E | 57530 | CGN | E | 6195 | RPS6KA1 | E |
| 7082 | TJP1 | E | 100506658 | OCLN | E | 1364 | CLDN4 | E | 27293 | SMPDL3B | E |
|  |  |  |  |  |  | 123920 | CMTM3 | M | 26154 | ABCA12 | E |
|  |  |  |  |  |  | 23019 | CNOT1 | E | 29984 | RHOD | E |
|  |  |  |  |  |  | 25927 | CNRIP1 | M | 3861 | KRT14 | E |
|  |  |  |  |  |  | 1300 | COL10A1 | M | 5583 | PRKCH | E |
|  |  |  |  |  |  | 1277 | COL1A1 | M | 79413 | ZBED2 | E |
|  |  |  |  |  |  | 1278 | COL1A2 | M | 11067 | C10ORF10 | E |
|  |  |  |  |  |  | 1281 | COL3A1 | M | 55227 | LRRC1 | E |
|  |  |  |  |  |  | 1289 | COL5A1 | M | 55620 | STAP2 | E |
|  |  |  |  |  |  | 1290 | COL5A2 | M | 3728 | JUP | E |
|  |  |  |  |  |  | 1291 | COL6A1 | M | 3566 | IL4R | E |
|  |  |  |  |  |  | 1292 | COL6A2 | M | 64065 | PERP | E |
|  |  |  |  |  |  | 1293 | COL6A3 | M | 9982 | FGFBP1 | E |
|  |  |  |  |  |  | 1295 | COL8A1 | M | 4642 | MYO1D | E |
|  |  |  |  |  |  | 1500 | CTNND1 | E | 2196 | FAT2 | E |
|  |  |  |  |  |  | 51339 | DACT1 | M | 23286 | WWC1 | E |
|  |  |  |  |  |  | 1783 | DYNC1LI2 | E | 7976 | FZD3 | E |
|  |  |  |  |  |  | 2014 | EMP3 | M | 7718 | ZNF165 | E |
|  |  |  |  |  |  | 2065 | ERBB3 | E | 6622 | SNCA | E |
|  |  |  |  |  |  | 54845 | ESRP1 | E | 79956 | KIAA1815 | E |
|  |  |  |  |  |  | 80004 | ESRP2 | E | 5652 | PRSS8 | E |
|  |  |  |  |  |  | 50848 | F11R | E | 10045 | SH2D3A | E |
|  |  |  |  |  |  | 2191 | FAP | M | 2774 | GNAL | E |
|  |  |  |  |  |  | 2200 | FBN1 | M | 638 | BIK | E |
|  |  |  |  |  |  | 2335 | FN1 | M | 1001 | CDH3 | E |
|  |  |  |  |  |  | 11167 | FSTL1 | M | 26049 | FAM169A | E |
|  |  |  |  |  |  | 2591 | GALNT3 | E | 3852 | KRT5 | E |
|  |  |  |  |  |  | 10082 | GPC6 | M | 2707 | GJB3 | E |
|  |  |  |  |  |  | 9289 | GPR56 | E | 9674 | KIAA0040 | E |
|  |  |  |  |  |  | 79977 | GRHL2 | E | 1952 | CELSR2 | E |
|  |  |  |  |  |  | 2995 | GYPC | M | 50848 | F11R | E |
|  |  |  |  |  |  | 51361 | HOOK1 | E | 54830 | NUP62CL | E |
|  |  |  |  |  |  | 5654 | HTRA1 | M | 1992 | SERPINB1 | E |
|  |  |  |  |  |  | 3624 | INHBA | M | 10653 | SPINT2 | E |
|  |  |  |  |  |  | 3664 | IRF6 | E | 10622 | POLR3G | E |
|  |  |  |  |  |  | 22801 | ITGA11 | M | 79767 | ELMO3 | E |
|  |  |  |  |  |  | 4017 | LOXL2 | M | 3557 | IL1RN | E |
|  |  |  |  |  |  | 131578 | LRRC15 | M | 10103 | TSPAN1 | E |
|  |  |  |  |  |  | 9053 | MAP7 | E | 10437 | IFI30 | E |
|  |  |  |  |  |  | 153562 | MARVELD2 | E | 5357 | PLS1 | E |
|  |  |  |  |  |  | 91862 | MARVELD3 | E | 28983 | TMPRSS11E | E |
|  |  |  |  |  |  | 4313 | MMP2 | M | 79098 | C1ORF116 | E |
|  |  |  |  |  |  | 253827 | MSRB3 | M | 247 | ALOX15B | E |
|  |  |  |  |  |  | 4645 | MYO5B | E | 1308 | COL17A1 | E |
|  |  |  |  |  |  | 4675 | NAP1L3 | M | 51750 | RTEL1 | E |
|  |  |  |  |  |  | 22795 | NID2 | M | 8771 | TNFRSF6B | E |
|  |  |  |  |  |  | 100506658 | OCLN | E | 3898 | LAD1 | E |
|  |  |  |  |  |  | 25903 | OLFML2B | M | 5774 | PTPN3 | E |
|  |  |  |  |  |  | 5118 | PCOLCE | M | 4486 | MST1R | E |
|  |  |  |  |  |  | 5159 | PDGFRB | M | 2041 | EPHA1 | E |
|  |  |  |  |  |  | 5376 | PMP22 | M | 1875 | E2F5 | E |
|  |  |  |  |  |  | 10631 | POSTN | M | 3854 | KRT6B | E |
|  |  |  |  |  |  | 5652 | PRSS8 | E | 6769 | STAC | E |
|  |  |  |  |  |  | 6678 | SPARC | M | 3691 | ITGB4 | E |
|  |  |  |  |  |  | 6692 | SPINT1 | E | 84830 | C6ORF105 | E |
|  |  |  |  |  |  | 6695 | SPOCK1 | M | 27165 | GLS2 | E |
|  |  |  |  |  |  | 23213 | SULF1 | M | 306 | ANXA3 | E |
|  |  |  |  |  |  | 23208 | SYT11 | M | 667 | DST | E |
|  |  |  |  |  |  | 7058 | THBS2 | M | 9938 | ARHGAP25 | E |
|  |  |  |  |  |  | 1462 | VCAN | M | 1824 | DSC2 | E |
|  |  |  |  |  |  | 7431 | VIM | M | 6535 | SLC6A8 | E |
|  |  |  |  |  |  | 9839 | ZEB2 | M | 51599 | LSR | E |
|  |  |  |  |  |  |  |  |  | 9076 | CLDN1 | E |
|  |  |  |  |  |  |  |  |  | 55638 | SYBU | E |
|  |  |  |  |  |  |  |  |  | 57834 | CYP4F11 | E |
|  |  |  |  |  |  |  |  |  | 894 | CCND2 | E |
|  |  |  |  |  |  |  |  |  | 2263 | FGFR2 | E |
|  |  |  |  |  |  |  |  |  | 3983 | ABLIM1 | E |
|  |  |  |  |  |  |  |  |  | 7498 | XDH | E |
|  |  |  |  |  |  |  |  |  | 816 | CAMK2B | E |
|  |  |  |  |  |  |  |  |  | 1830 | DSG3 | E |
|  |  |  |  |  |  |  |  |  | 4671 | NAIP | E |
|  |  |  |  |  |  |  |  |  | 100506658 | OCLN | E |
|  |  |  |  |  |  |  |  |  | 3872 | KRT17 | E |
|  |  |  |  |  |  |  |  |  | 6289 | SAA2 | E |
|  |  |  |  |  |  |  |  |  | 79056 | PRRG4 | E |
|  |  |  |  |  |  |  |  |  | 288 | ANK3 | E |
|  |  |  |  |  |  |  |  |  | 10974 | C10ORF116 | E |
|  |  |  |  |  |  |  |  |  | 56649 | TMPRSS4 | E |
|  |  |  |  |  |  |  |  |  | 1474 | CST6 | E |
|  |  |  |  |  |  |  |  |  | 10397 | NDRG1 | E |
|  |  |  |  |  |  |  |  |  | 6279 | S100A8 | E |
|  |  |  |  |  |  |  |  |  | 11151 | CORO1A | E |
|  |  |  |  |  |  |  |  |  | 25818 | KLK5 | E |
|  |  |  |  |  |  |  |  |  | 23086 | EXPH5 | E |
|  |  |  |  |  |  |  |  |  | 50805 | IRX4 | E |
|  |  |  |  |  |  |  |  |  | 3664 | IRF6 | E |
|  |  |  |  |  |  |  |  |  | 51361 | HOOK1 | E |
|  |  |  |  |  |  |  |  |  | 9048 | ARTN | E |
|  |  |  |  |  |  |  |  |  | 10367 | MICU1 | E |
|  |  |  |  |  |  |  |  |  | 56606 | SLC2A9 | E |
|  |  |  |  |  |  |  |  |  | 11202 | KLK8 | E |
|  |  |  |  |  |  |  |  |  | 55287 | TMEM40 | E |
|  |  |  |  |  |  |  |  |  | 23650 | TRIM29 | E |
|  |  |  |  |  |  |  |  |  | 1839 | HBEGF | E |
|  |  |  |  |  |  |  |  |  | 220 | ALDH1A3 | E |
|  |  |  |  |  |  |  |  |  | 80004 | ESRP2 | E |
|  |  |  |  |  |  |  |  |  | 55930 | MYO5C | E |
|  |  |  |  |  |  |  |  |  | 1594 | CYP27B1 | E |
|  |  |  |  |  |  |  |  |  | 3553 | IL1B | E |
|  |  |  |  |  |  |  |  |  | 10874 | NMU | E |
|  |  |  |  |  |  |  |  |  | 3868 | KRT16 | E |
|  |  |  |  |  |  |  |  |  | 999 | CDH1 | E |
|  |  |  |  |  |  |  |  |  | 3714 | JAG2 | E |
|  |  |  |  |  |  |  |  |  | 7447 | VSNL1 | E |
|  |  |  |  |  |  |  |  |  | 6019 | RLN2 | E |
|  |  |  |  |  |  |  |  |  | 1515 | CTSL2 | E |
|  |  |  |  |  |  |  |  |  | 6850 | SYK | E |
|  |  |  |  |  |  |  |  |  | 6288 | SAA1 | E |
|  |  |  |  |  |  |  |  |  | 54566 | EPB41L4B | E |
|  |  |  |  |  |  |  |  |  | 79589 | RNF128 | E |
|  |  |  |  |  |  |  |  |  | 6768 | ST14 | E |
|  |  |  |  |  |  |  |  |  | 55214 | LEPREL1 | E |
|  |  |  |  |  |  |  |  |  | 5266 | PI3 | E |
|  |  |  |  |  |  |  |  |  | 10053 | AP1M2 | E |
|  |  |  |  |  |  |  |  |  | 548596 | CKMT1A | E |
|  |  |  |  |  |  |  |  |  | 1159 | CKMT1B | E |
|  |  |  |  |  |  |  |  |  | 79977 | GRHL2 | E |
|  |  |  |  |  |  |  |  |  | 23779 | ARHGAP8 | E |
|  |  |  |  |  |  |  |  |  | 553158 | PRR5-ARHGAP8 | E |
|  |  |  |  |  |  |  |  |  | 3485 | IGFBP2 | E |
|  |  |  |  |  |  |  |  |  | 3606 | IL18 | E |
|  |  |  |  |  |  |  |  |  | 768 | CA9 | E |
|  |  |  |  |  |  |  |  |  | 57402 | S100A14 | E |
|  |  |  |  |  |  |  |  |  | 760 | CA2 | E |
|  |  |  |  |  |  |  |  |  | 3866 | KRT15 | E |
|  |  |  |  |  |  |  |  |  | 10205 | EVA1 | E |
|  |  |  |  |  |  |  |  |  | 161291 | TMEM30B | E |
|  |  |  |  |  |  |  |  |  | 6278 | S100A7 | E |
|  |  |  |  |  |  |  |  |  | 5650 | KLK7 | E |
|  |  |  |  |  |  |  |  |  | 3963 | LGALS7 | E |
|  |  |  |  |  |  |  |  |  | 10468 | FST | E |
|  |  |  |  |  |  |  |  |  | 1525 | CXADR | E |
|  |  |  |  |  |  |  |  |  | 6590 | SLPI | E |
|  |  |  |  |  |  |  |  |  | 54845 | RBM35A | E |
|  |  |  |  |  |  |  |  |  | 57111 | RAB25 | E |
|  |  |  |  |  |  |  |  |  | 7345 | UCHL1 | E |
|  |  |  |  |  |  |  |  |  | 5655 | KLK10 | E |
|  |  |  |  |  |  |  |  |  | 4072 | TACSTD1 | E |
|  |  |  |  |  |  |  |  |  | 5055 | SERPINB2 | E |
|  |  |  |  |  |  |  |  |  | 6698 | SPRR1A | E |
|  |  |  |  |  |  |  |  |  | 2261 | FGFR3 | E |
|  |  |  |  |  |  |  |  |  | 6699 | SPRR1B | E |
|  |  |  |  |  |  |  |  |  | 10516 | FBLN5 | M |
|  |  |  |  |  |  |  |  |  | 26585 | GREM1 | M |
|  |  |  |  |  |  |  |  |  | 1281 | COL3A1 | M |
|  |  |  |  |  |  |  |  |  | 1278 | COL1A2 | M |
|  |  |  |  |  |  |  |  |  | 1634 | DCN | M |
|  |  |  |  |  |  |  |  |  | 1000 | CDH2 | M |
|  |  |  |  |  |  |  |  |  | 5168 | ENPP2 | M |
|  |  |  |  |  |  |  |  |  | 10631 | POSTN | M |
|  |  |  |  |  |  |  |  |  | 5999 | RGS4 | M |
|  |  |  |  |  |  |  |  |  | 9315 | C5ORF13 | M |
|  |  |  |  |  |  |  |  |  | 5396 | PRRX1 | M |
|  |  |  |  |  |  |  |  |  | 2200 | FBN1 | M |
|  |  |  |  |  |  |  |  |  | 5552 | SRGN | M |
|  |  |  |  |  |  |  |  |  | 6695 | SPOCK1 | M |
|  |  |  |  |  |  |  |  |  | 51334 | PRR16 | M |
|  |  |  |  |  |  |  |  |  | 10395 | DLC1 | M |
|  |  |  |  |  |  |  |  |  | 274 | BIN1 | M |
|  |  |  |  |  |  |  |  |  | 23179 | RGL1 | M |
|  |  |  |  |  |  |  |  |  | 3487 | IGFBP4 | M |
|  |  |  |  |  |  |  |  |  | 25945 | PVRL3 | M |
|  |  |  |  |  |  |  |  |  | 1009 | CDH11 | M |
|  |  |  |  |  |  |  |  |  | 56944 | OLFML3 | M |
|  |  |  |  |  |  |  |  |  | 4313 | MMP2 | M |
|  |  |  |  |  |  |  |  |  | 10398 | MYL9 | M |
|  |  |  |  |  |  |  |  |  | 1290 | COL5A2 | M |
|  |  |  |  |  |  |  |  |  | 1490 | CTGF | M |
|  |  |  |  |  |  |  |  |  | 10979 | PLEKHC1 | M |
|  |  |  |  |  |  |  |  |  | 6935 | ZEB1 | M |
|  |  |  |  |  |  |  |  |  | 4919 | ROR1 | M |
|  |  |  |  |  |  |  |  |  | 5732 | PTGER2 | M |
|  |  |  |  |  |  |  |  |  | 1123 | CHN1 | M |
|  |  |  |  |  |  |  |  |  | 5376 | PMP22 | M |
|  |  |  |  |  |  |  |  |  | 9697 | TRAM2 | M |
|  |  |  |  |  |  |  |  |  | 6876 | TAGLN | M |
|  |  |  |  |  |  |  |  |  | 7130 | TNFAIP6 | M |
|  |  |  |  |  |  |  |  |  | 90993 | CREB3L1 | M |
|  |  |  |  |  |  |  |  |  | 7358 | UGDH | M |
|  |  |  |  |  |  |  |  |  | 3037 | HAS2 | M |
|  |  |  |  |  |  |  |  |  | 11080 | DNAJB4 | M |
|  |  |  |  |  |  |  |  |  | 1031 | CDKN2C | M |
|  |  |  |  |  |  |  |  |  | 80212 | CCDC92 | M |
|  |  |  |  |  |  |  |  |  | 7474 | WNT5A | M |
|  |  |  |  |  |  |  |  |  | 3486 | IGFBP3 | M |
|  |  |  |  |  |  |  |  |  | 8493 | PPM1D | M |
|  |  |  |  |  |  |  |  |  | 11259 | FILIP1L | M |
|  |  |  |  |  |  |  |  |  | 56034 | PDGFC | M |
|  |  |  |  |  |  |  |  |  | 6926 | TBX3 | M |
|  |  |  |  |  |  |  |  |  | 64131 | XYLT1 | M |
|  |  |  |  |  |  |  |  |  | 2191 | FAP | M |
|  |  |  |  |  |  |  |  |  | 1805 | DPT | M |
|  |  |  |  |  |  |  |  |  | 6781 | STC1 | M |
|  |  |  |  |  |  |  |  |  | 3887 | KRT81 | M |
|  |  |  |  |  |  |  |  |  | 4312 | MMP1 | M |
|  |  |  |  |  |  |  |  |  | 9956 | HS3ST2 | M |
|  |  |  |  |  |  |  |  |  | 29995 | LMCD1 | M |
|  |  |  |  |  |  |  |  |  | 84656 | N-PAC | M |
|  |  |  |  |  |  |  |  |  | 23157 | SEPT6 | M |
|  |  |  |  |  |  |  |  |  | 7025 | NR2F1 | M |
|  |  |  |  |  |  |  |  |  | 51097 | SCCPDH | M |
|  |  |  |  |  |  |  |  |  | 79083 | MLPH | M |
|  |  |  |  |  |  |  |  |  | 4053 | LTBP2 | M |
|  |  |  |  |  |  |  |  |  | 7168 | TPM1 | M |
|  |  |  |  |  |  |  |  |  | 25959 | ANKRD25 | M |
|  |  |  |  |  |  |  |  |  | 4921 | DDR2 | M |
|  |  |  |  |  |  |  |  |  | 9037 | SEMA5A | M |
|  |  |  |  |  |  |  |  |  | 7041 | TGFB1I1 | M |
|  |  |  |  |  |  |  |  |  | 5118 | PCOLCE | M |
|  |  |  |  |  |  |  |  |  | 90627 | STARD13 | M |
|  |  |  |  |  |  |  |  |  | 4811 | NID1 | M |
|  |  |  |  |  |  |  |  |  | 81493 | SYNC1 | M |
|  |  |  |  |  |  |  |  |  | 55068 | ENOX1 | M |
|  |  |  |  |  |  |  |  |  | 11167 | FSTL1 | M |
|  |  |  |  |  |  |  |  |  | 7431 | VIM | M |
|  |  |  |  |  |  |  |  |  | 4311 | MME | M |
|  |  |  |  |  |  |  |  |  | 219654 | C10ORF56 | M |
|  |  |  |  |  |  |  |  |  | 4052 | LTBP1 | M |
|  |  |  |  |  |  |  |  |  | 8829 | NRP1 | M |
|  |  |  |  |  |  |  |  |  | 7070 | THY1 | M |
|  |  |  |  |  |  |  |  |  | 10529 | NEBL | M |
|  |  |  |  |  |  |  |  |  | 64759 | TNS3 | M |
|  |  |  |  |  |  |  |  |  | 1893 | ECM1 | M |
|  |  |  |  |  |  |  |  |  | 2192 | FBLN1 | M |
|  |  |  |  |  |  |  |  |  | 7846 | TUBA1A | M |
|  |  |  |  |  |  |  |  |  | 51226 | COPZ2 | M |
|  |  |  |  |  |  |  |  |  | 79901 | CYBRD1 | M |
|  |  |  |  |  |  |  |  |  | 8613 | PPAP2B | M |
|  |  |  |  |  |  |  |  |  | 5806 | PTX3 | M |
|  |  |  |  |  |  |  |  |  | 9415 | FADS2 | M |
|  |  |  |  |  |  |  |  |  | 633 | BGN | M |
|  |  |  |  |  |  |  |  |  | 10194 | TSHZ1 | M |
|  |  |  |  |  |  |  |  |  | 253461 | ZBTB38 | M |
