# Supplementary material for: An individualized transcriptional signature to predict the epithelial-mesenchymal transition based on relative expression ordering
Source: Aging (Albany NY). 2020 Jul 8;12(13):13172–86. doi: 10.18632/aging.103407 (PMC7377874; doi:10.18632/aging.103407)
Supplement: Supplementary Table 2 [file aging-12-103407-s002..pdf]

SUPPLEMENTARY TABLES

Supplementary Table 2. Expression of 20 immune checkpoint genes in TCGA OvCa dataset.

Gene symbols	Entrez ID	P-value	Trend
ADORA2A	135	0.27	
CCL2	6347	3.17e-11	√
PDL1	29126	-	-
CD276	80381	-	-
CD4	920	1.64e-07	√
CXCR4	7852	0.17	√
CTLA4	1493	0.045	
HAVCR2	84868	-	-
ICOS	29851	0.0091	√
ICOSLG	23308	0.0054	
IL1A	3552	0.53	√
IL6	3569	1.46E-13	√
IL10	3586	2.59E-05	√
LAG3	3902	0.23	
PD1	5133	0.036	
PDL2	80380	0.0093	√
TGFB1	7040	1.58e-17	√
TNFRSF4	7293	0.026	√
TNFSF4	7292	2.17e-31	√
TNFRSF9	3604	2.25e-05	√

Note: Differentially expressed analysis of 20 immune checkpoint genes between TCGA mesenchymal and epithelial OvCa samples were determined by Two-sample Wilcoxon rank-sum test. *P*-values were corrected using the Benjamini and Hochberg approach; Trend, "√" represents the gene is highly expressed in mesenchymal samples (vs. epithelial), which is consistent with expectations, otherwise it is marked with null. "-" represents the gene has not been detected in the TCGA OvCa cohort.
